# Identification of yeast cell cycle regulated genes based on genomic features

**DOI:** 10.1186/1752-0509-7-70

**Published:** 2013-07-29

**Authors:** Chao Cheng, Yao Fu, Linsheng Shen, Mark Gerstein

**Affiliations:** 1 Department of Genetics, Geisel School of Medicine at Dartmouth, Hanover, NH 03755, USA; 2 Institute for Quantitative Biomedical Sciences, Norris Cotton Cancer Center, Geisel School of Medicine at Dartmouth, Lebanon, NH 03766, USA; 3 Program in Computational Biology and Bioinformatics, Yale University, 260 Whitney Avenue, New Haven, CT 06520, USA; 4 Department of Biological Sciences, Purdue University, West Lafayette, IN 47907, USA; 5 Department of Molecular Biophysics and Biochemistry, Yale University, 260 Whitney Avenue, New Haven, CT 06520, USA; 6 Department of Computer Science, Yale University, 260 Whitney Avenue, New Haven, CT 06520, USA

**Keywords:** Cell cycle regulated genes, Genomic features, Prediction

## Abstract

**Background:**

Time-course microarray experiments have been widely used to identify cell cycle regulated genes. However, the method is not effective for lowly expressed genes and is sensitive to experimental conditions. To complement microarray experiments, we propose a computational method to predict cell cycle regulated genes based on their genomic features – transcription factor binding and motif profiles.

**Results:**

Through integrating gene-expression data with ChIP-chip binding and putative binding sites of transcription factors, our method shows high accuracy in discriminating yeast cell cycle regulated genes from non-cell cycle regulated ones. We predict 211 novel cell cycle regulated genes. Our model rediscovers the main cell cycle transcription factors and provides new insights into the regulatory mechanisms. The model also reveals a regulatory circuit mediated by a number of key cell cycle regulators.

**Conclusions:**

Our model suggests that the periodical pattern of cell cycle genes is largely coded in their promoter regions, which can be captured by motif and transcription factor binding data. Cell cycle is controlled by a relatively small number of master transcription factors. The concept of genomic feature based method can be readily extended to human cell cycle process and other transcriptionally regulated processes, such as tissue-specific expression.

## Background

Cell division is under precise regulation in eukaryotic organisms. Many genes functioning in cell division are regulated and expressed right before they are needed [1]. These genes show periodical expression patterns with peaks at certain mitotic stages. The most effective way for identifying these genes is to analyze gene expression profiles using time course microarray. For example, in *Saccharomyces cerevisiae*, several hundreds of periodically expressed genes have been identified based on microarray [2-6]. Despite the high throughput, microarray has its limitations in terms of identifying cell cycle regulated gene. First, genes identified from different studies are poorly overlapped, presumably due to varied experimental conditions or de-synchronization problems [7]. Second, it is often difficult to identify lowly expressed or weakly regulated genes due to technical issues [4,7].

To overcome the limitations of microarray experiments, several computational methods have been proposed as compensatory methods to find cell cycle regulated genes in yeast. Streib et al. [8] predicted cell cycle regulated genes based on causal interaction using regulatory networks. Wang et al. [9] combined genetic interactions and co-expression data to infer candidate cell cycle regulated genes. These two methods are based on the assumption that these genes tend to possess interacting relationship with each other. Alternatively, de Lichtenberg et al. found that many cell cycle regulated genes shared common protein features, thus proposed a method to predict novel ones based on protein features [7]. These protein-level features mainly reflect cell cycle regulation at the post-translational level, such as protein stability. However, the DNA sequence features, which contribute at the transcriptional level, are completely ignored by this method.

A subset of transcription factors responsible for the coordinated regulation of cell cycle genes has been uncovered [10]. For example, Mbp1 is a crucial transcription factor involved in cell cycle progression from G1 to S phase [11]; Mcm1/Fkh1/Ndd1 cooperatively regulate G2/M genes through binding to their promoters [12]. Since cell cycle regulated genes tend to be regulated by a common set of regulators, we hypothesize that whether a gene is cell cycle regulated can be inferred based on genomic features - specifically, the regulatory transcriptional factors (TFs) binding (trans-factors) and motifs profiles in their promoters (cis-elements). Fortunately, in yeast the genomic occupations of most TFs have been identified with ChIP-chip experiment [13]. Meanwhile, the potential regulatory cis-elements have been systematically investigated by computational analysis [14]. Thus, we are motivated to construct statistical models to predict yeast cell cycle regulated genes based on these features.

In this study, we combined the large-scale TF binding data and motif profiles to predict cell cycle regulated genes in budding yeast. The predictive model achieved high accuracy evaluated by 10-fold cross-validations. We predicted 211 novel cell cycle genes and determined their potential phases (G1, S, G2, or M phase). These genes are enriched for cell cycle related processes according to Gene Ontology (GO) analysis and tend to be lowly expressed. The model also provided us a set of TFs significantly contributed to cell cycle gene regulation. Our analysis in this work suggests that genomic features are informative for predicting periodically expressed genes. The statistical model proposed in this paper can be applied to identify cell cycle regulated genes in other organisms or broadly to genes under transcriptional level regulation.

## Results

### Genomic features are predictive of cell cycle regulated genes

**Figure 1:**
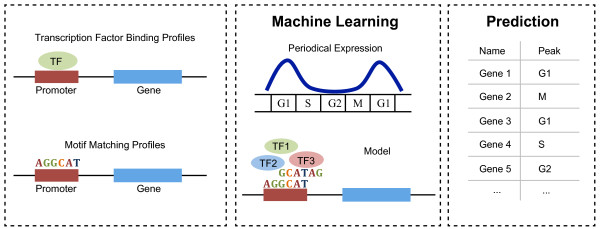
**Schematic description of genomic feature based method.** For each gene, 203 transcription factors binding and 537 motif-matching profiles were collected to train the prediction model. The model was then applied to quantify the periodicity and stage specificity of yeast genes.

Considering the discrepancy of different experimental studies, we chose 599 periodically expressed genes (hereafter called cell cycle genes) and 454 non-periodically expressed ones (hereafter called non-cell cycle genes) as our training data (see Methods for details) based on the meta-analysis in Cyclebase.org [15] that combines multiple yeast cell cycle time-course datasets. To examine whether transcriptional level regulation can explain the periodical expression patterns, we focused our analysis on proximal genomic features: trans-features, TF binding data from ChIP-chip experiments, and cis-features, motifs profiles in gene promoter regions (Figure 1).

**Figure 2:**
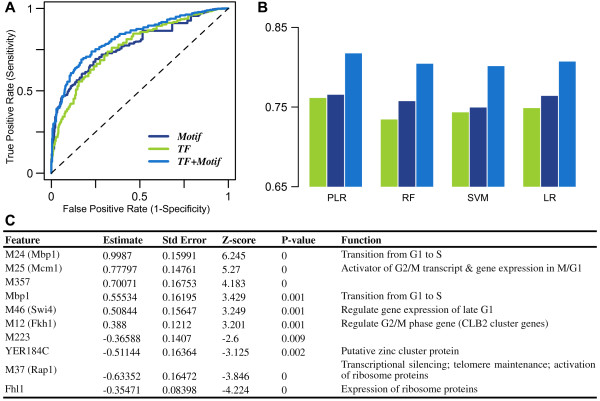
**Performance of prediction methods. (A)** ROC curves for TF binding, motif, and TF + Motif combined models using penalized logistic regression with 10-fold cross validations. **(B)** AUC comparisons of four different machine learning methods, penalized logistic regression (PLR), random forest (RF), support vector machine (SVM) and ordinary logistic regression (LR). **(C)** Significant genomics features discriminating cell cycle regulated genes from non-cell cycle regulated ones (p-value < 0.01).

Specifically, for each gene we collected the binding strength of 203 TFs from Harbison et al*.*[13] and calculated the matching score of 537 motifs from Beer et al. [14] (see Methods for details). A penalized logistic regression model (PLR with L2 norm) was then fitted to classify cell cycle genes using these genomic features. We evaluated the performance of the model using 10-fold cross-validations (see Methods). As shown in Figure 2A, both the TF model (with 34 pre-selected TF binding predictors) and the motif model (with 21 pre-selected motif predictors) achieved fairly high accuracies with AUC (area under ROC curves, see Methods) scores of 0.762 and 0.766, respectively. Moreover, the combined TF + Motif model achieved the highest predictive accuracy (AUC = 0.818). These results indicate that cell cycle and non-cell cycle genes are different in their transcriptional regulation, which can be reflected by both TF binding and motif existence in their promoters. They also indicate that the two types of genomic features provide, at least partially, regulatory information from different perspectives.

In addition to the PLR model, we also evaluated the performance of other methods, including Logistic Regression (LR), Random Forest (RF) and Support Vector Machine (SVM). For all methods, the TF + Motif model gave rise to higher accuracy than the TF or motif only models (Figure 2B). PLR model has slightly better performance and is easy to be interpreted. Thus, we focused on results from this method in the subsequent analysis, but similar conclusions can be achieved when other methods are used (Additional file 1).

We then investigated key genomic features that were critical for discriminating cell cycle genes from non-cell cycle ones. Significant features (p-value <0.01) in the combined model are listed in Figure 2C. Positive contributors (z-score > 0) include the trans-feature Mbp1 and several cis-features, i.e. the motifs for Mbp1, Mcm1, Swi4 and Fkh1. All of these TFs are known to be important for cell cycle regulation. Mbp1 is the DNA binding component of MBF complex, which binds to the promoters of DNA synthesis genes and regulates gene expression during G1/S transition [11,16-18]. Mcm1, together with Fkh2 or Fkh1, activates G2/M expressed genes through the recruitment of Ndd1 [10,12,18]. It is also involved in the repression of M/G1 gene transcription by interacting with Yox1p and Yhp1p [18,19]. Swi4 shares a similar function with Mbp1. It regulates late G1 specific target genes by forming an SBF complex with Swi6 [3,20-25]. Motif M357 is a significant predictor for cell cycle genes as identified by the model. The regulatory protein binding with this motif is not clear, but it shares certain similarity with Mbp1 or Swi6 binding motifs (TOMTOM [26], p-values ~4e-4). The model also identifies several negative contributors (z-score < 0), e.g. Fhl1 and the binding motif of Rap1. These two TFs are involved in transcriptional regulation of ribosomal proteins, which are constitutively expressed at very high levels during cell growth [27,28]. Generally, genes associated with positive features are more likely to be periodically expressed; conversely, genes associated with negative features are less likely to be periodical.

### Genomic features distinguish phase-specific cell cycle genes

**Figure 3:**
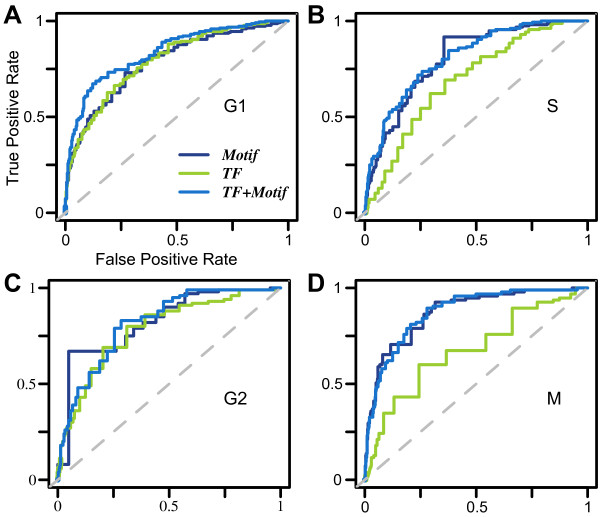
**Phase specific models (selected phase vs. all other phases). (A)** G1 phase specific. **(B)** S phase specific. **(C)** G2 phase specific. **(D)** M phase specific.

Based on the expression peak time, cell cycle genes can be further divided into G1, S, G2 and M phase genes. We examined whether phase specific genes had unique genomic features that distinguish them from other periodical ones. Among the 599 cell cycle genes, 219, 156, 100 and 95 were classified as G1, S, G2 and M phase specific genes, respectively, based on Cyclebase [15]. We constructed four phase-specific models to distinguish genes in one phase from the other phases with 10-fold cross-validations. As shown in Figure 3, genomic features are also predictive of phase-specific cell cycle genes with AUC scores of G1, S, G2, and M phases 0.833, 0.803, 0.807 and 0.868, respectively. Although phase classification is somehow arbitrary, the high AUC scores indicate that these genomic features are informative to infer phase-specificity of genes. In Table 1, we listed the significant genomic features in at least one cell cycle phase. Transcriptional regulation of genes at a certain phase is mainly controlled by a small number of TFs or cis-regulatory motifs. Most of the significant features are previously known to be cell cycle stage specific regulators. For example, Mbp1 is the major regulator in late G1 phase and Swi5 activates gene transcription at the M/G1 checkpoint [29]. Consistently, our model suggests that cell cycle genes associated with Swi5 or Mbp1 binding motifs are more likely to be G1 specific genes. In general, the positive features in one phase often contribute negatively in other phases due to the exclusive nature of these phase-specific models.

**Table 1:** Significant genomic features in phase specific models

**TF or Motif**	**Function**	**G1**	**S**	**G2**	**M**
**Mbp1**	Late	+**		_**	
**Swi5**	M/G1, G1	+**		_**	
**M24**	Mbp1, late G1	+**		_**	_**
M496		+**			
M568		+*			
M571		+*			
**Ndd1**	G2/M	_**		+*	
**Fkh2**	G2/M	_**		+*	
M493		_**			
Dot6	De-acetylation	_**			
M6	Bas1, purine and hisitdine biosynthesis, meiotic recombination	_*			
Met32	Methionine-biosynthesis	_*			
M207		_*			
Srd1	Pre-rRNA processing	_*			
**M12**	Fkh1, G2/M		+**		_**
**M46**	Swi4, late G1		+**	_**	_**
**M95**	Met32 ′		+**		_**
M93			+*		
**M25**	Mcm1, G2/M, M/G1		_**		+**
Ace2	M/G1		_**		
M374			_**		
M524			_**		
Rap1	Telomere related		_*		
M203			_*		
YPR196W	Maltose response		_*		
M103			_*		
Stp4			_*		
M577				_*	
M357				_*	
M179				_*	
Met31	Methionine-biosynthesis				_*
M168					_*

“+” denotes positive contributors; “-” means negative contributors; “**” means P value < 0.01; “*” means P value < 0.05. M95 is predicted to be Met32 binding motif. Genomic features in more than one stage are highlighted in bold.

Phase-specific models suggest some interesting findings about regulatory mechanisms. The model indicates Fkh1 as a S-phase predictor, although it is mainly known to function at the G2/M checkpoint. This is supported by Simon et al. [10] showing that many S phase genes contain Fkh1 binding sites in their promoter regions. Although Mbp1 and Swi4 are previously known as late G1 regulators, our analysis suggests that Swi4 might function later than Mbp1. In Cyclebase, Mbp1 reaches its expression peak during S/G2 stages, while Swi4 demonstrates peak in middle G1 phase. Consistently, motif analysis suggests that Swi4 is likely to be regulated by Mbp1. The unexpected predictor for S-phase genes is M95, which resembles Met32 binding motif (AAACTGTGG) (TOMTOM [26]). Met32 is generally known as a transcriptional regulator of methionine biosynthetic genes [30], but recently has been shown as the primary mediator of the Met30-controlled cell cycle checkpoint [31]. Spellman et al. [3] also found that several S-phase genes function in methionine metabolism and contain a Met32/Met31 motif in their promoters. Our analysis suggests a crosstalk between cell cycle control and metabolic regulation.

### Novel cell cycle regulated genes are identified by genomic feature based method

**Figure 4:**
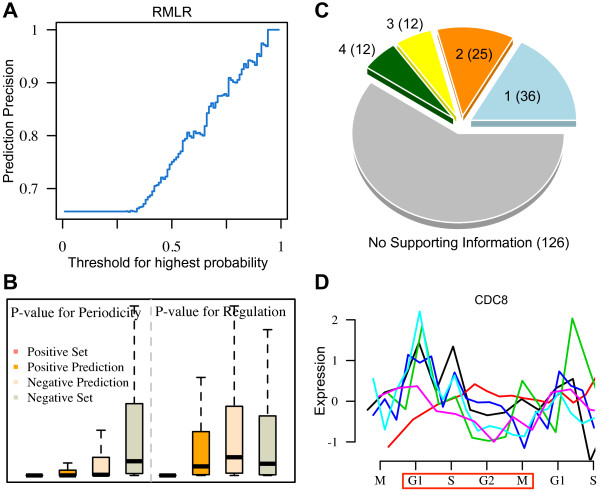
**Multiple phase prediction model and cell cycle gene prediction. (A)** Performance of regularized multinomial logistic regression (RMLR) in separating phase specific cell cycle genes. **(B)** PPer (p value for periodical) and PReg (p value for regulation) for different gene sets: known cell cycle gene set, predicted cell cycle gene set, predicted non-cell cycle gene set and known non-cell cycle gene set. **(C)** Support from other studies. **(D)** Expression profiles of CDC8 from 6 experiments in Cyclebase. Red rectangle denotes the second induced cell cycle.

We applied the TF + Motif model to predict novel periodically expressed genes. After excluding training set, we calculated the probability of genes to be cell cycle regulated. Among the 5,168 unclassified genes, 211 have a probability greater than 0.8 (corresponding to a false positive rate of 2.86% and a sensitivity of 37.7%). We then inferred their phase-specificity using penalized multinomial logistic regression (RMLR, see Methods for details). As a multi-class classification method, RMLR simultaneously calculates the probabilities of a gene to be G1, S, G2 or M phase specific, and assigns the gene to the phase with highest probability. One can set a threshold for the highest probability to improve the prediction accuracy, i.e. assign a gene to be “unclear” if the highest probability is lower than the threshold. The performance of the RMLR model was evaluated using the positive training data (576 cell cycle genes) with 10 fold cross-validations, resulting in a multi-class AUC score [32] of 0.885 (see Methods for multiple class AUC score calculation). Without setting threshold for highest probability, about 65.7% of genes were correctly assigned to their peak stages; increase of the threshold led to rapid precision improvement (Figure 4A).

Among the 211 predicted genes, 99, 52, 41 and 19 were assigned to G1, S, G2, M phases, respectively, according to the maximum probability they achieved in the four phases (Additional file 2). The higher the maximum probability, the more likely the phase is correctly assigned. These genes tend to have lower p-values in both periodicity (PPer, the significance of periodical expression pattern in the cell cycle) and regulation (PReg, the significance of varied expression levels in the cell cycle) than the non-cell cycle genes (detailed definition of these p values, refer to Cyclebase [15]). As shown in Figure 4B, cell cycle regulated genes in training set (positive set) have lowest PPer and PReg as expected, whereas non-cell cycle genes (negative set) have significantly higher values (p-value < 2.2e-16, wilcoxon test). The 211 novel genes have significant lower PPer and PReg compared to predicted non-cell cycle genes (p-value = 8e-7 for PPer and p-value = 3e-5 for PReg, wilcoxon test). These results indicate that these predicted genes tend to have periodical expression patterns and varied expression levels across the cell cycle. Consistent with previous assumptions, our predicted genes have more physical interactions (BIOGRID [33]) with known cell cycle genes compared to those that are predicted not to be (p-value < 4e-4) (see Methods).

We collected 9 experimental or predictive results from previous studies (see Methods for details). Out of the 211 predicted genes, we found that 85 (40.3%) genes were supported by at least one evidence, as shown in Figure 4C (Additional file 3). Specifically, 24 genes have been shown as periodical ones in at least one experiment in Cyclebase [2-5,15]; 33 are in the cell cycle gene list identified by Granovskaia et al. [34]; and 60 are reported as such by Rowicka et al. [35]. In contrast, only 18.7% of genes in the negative prediction set have been supported by these data sources (p-value =3e-8, fisher’s exact test). A recent study in fission yeast identified 513 cell cycle genes with long cell phenotype using knockout [36]. We examined the cell cycle orthologs of the 211 predicted cell cycle genes. Out of the 64 genes with orthologs in fission yeast, 9 (14%) are identified as cell cycle ones, with only slightly higher percentage than the predicted non-cell cycle genes (13%). It has been shown that the overlap of periodically expressed genes between budding and fission yeast is very small [37]. However, the predicted cell cycle genes with orthologs that are also cell cycle related in fission yeast might be core cell cycle genes (we added those genes in Additional file 3).

We investigated the functions of these 211 predicted cell cycle genes using Gene Ontology and Saccharomyces Genome Database (SGD) [38]. These genes are involved in a wide range of biological functions. Based on SGD, 50 genes participate in unknown biological process and the others are involved in lipid metabolic process, response to chemical stimulus, mitotic cell cycle, cytoskeleton organization and so on (see Additional file 2 for detailed gene description). GO analysis from DAVID [39] (see Methods) suggests the enrichment of genes related to cell walls, organelle fission, chromosome condensation, DNA packaging, nuclear localization and other processes. More specifically, the predicted G1-specific genes are mostly involved in cellular metabolism, such as lipid metabolic process, cellular protein catabolic process, cytoskeleton organization and carbohydrate metabolic process. This is consistent with the concept that G1 phase is the interval with enhanced protein synthesis and metabolism preparing for mitosis. For the predicted S-specific genes, many are involved in cytoskeleton organization, mitotic cell cycle, organelle fission, chromosome segregation, and regulation of organelle organization. G2 phase is another intermediate phase preparing for mitotic process. Consistently, the predicted G2-specific genes function in lipid metabolic process, carbohydrate metabolic process and other metabolic pathways. They are also involved in regulation of cell cycle, sporulation, and trans-membrane transportation. The predicted M-specific genes are mainly involved in nuclear related processes, nuclear transport, response to DNA damage/chemical stimulus, signaling and DNA repair. Considering the complexity of cell cycle process and the intensive regulatory cooperation between different layers, it is not surprising to observe such a high diversity in functions of the predicted cell cycle genes (see Additional file 4 for results of GO analysis).

For those predicted genes coding for transcription factors, we investigated their transcriptional regulators based on the YEASTRACT database (Yeast Search for Transcriptional Regulators And Consensus Tracking) [40], which provides a curated repository of regulatory associations between transcription factors (TF) and potential target genes in yeast. We found that many of these predicted cell cycle TFs themselves were regulated by the known cell cycle regulators. For instance, Cyc8, Mga1, Haa1, Hms2, Mot3 and Tye7 are regulated by Mbp1/Swi4; Yap6, Rsf2, Ume1 and Ime1 are regulated by Fkh1/Fkh2; Tfc4, Ime1, Hms2, Tye7 and Zds2 are regulated by the M phase regulator, Mcm1/Ace2. These TFs might function downstream of key cell cycle TFs to regulate the transcription of a specific group of genes. For example, Ime1 is required for sporulation and has been shown as one of the major regulators that induce meiosis [41]. In consistency with this function, our model predicts Ime1 to be a cell cycle gene of G2/M specific.

We also investigated the question: why these predicted genes were not identified as such by the meta-analysis in Cyclebase. We found that this was mostly due to high noise of microarray data or loss of synchronization during the cell cycle induction. For example, we predicted Cdc8 as a G1-specific gene with a probability of 0.73. It contains a strong Mbp1 binding motif (M24) in its promoter region. Although Cdc8 is identified as a periodically expressed gene by one of previous studies [42], it has only moderately significant overall P-value (p-value > 0.001) in its periodical expression patterns according to Cyclebase, and thus was not included in our positive set. We examined the expression profiles of Cdc8 in the 6 experiments used by Cyclebase. As shown in Figure 4D, it turns out that 5 of these profiles demonstrate a potential peak at the G1 phase; however, the peak disappears after the second cell cycle, presumably due to de-synchronization of cells. Another example is the serine/threonine protein kinase, Cla4, which is involved in mitotic exit and cytokinesis. Our model predicts Cla4 as a cell cycle regulated gene with peak expression at M/G1. It shows strong binding affinity with Mcm1, Swi4 (motif existence) and Mbp1 (TF binding) in its promoter. Binding of Mcm1 and Swi4 with this gene is also verified by experiments [10]. In consistency with our prediction, the microarray expression profiles of Cla4 demonstrate a peak at M/G1. However, it was not identified as a cell cycle gene, apparently due to the loss of periodically expression pattern from the second cell cycle (Additional file 5).

**Figure 5:**
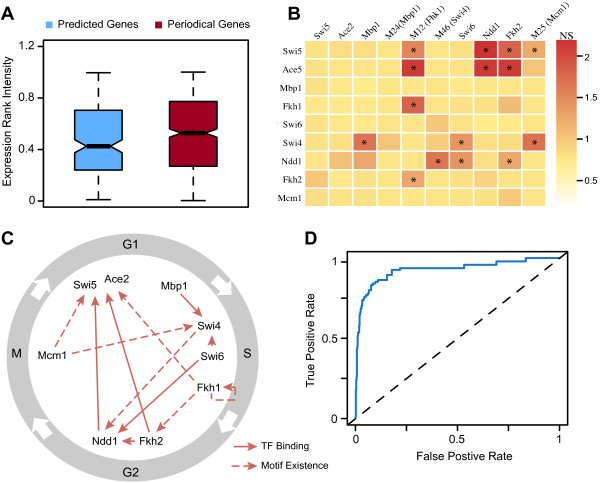
**Regulatory circuit of cell cycle transcription factors. (A)** mRNA expression intensity of newly predicted and known cell cycle genes. **(B)** Normalization scores (NS) of key genomic features (both transcription factor binding scores and motif matching scores) in promoters of 9 key cell cycle transcription factors. Asterisk denotes regulatory relationship passing certain criteria (see Methods). **(C)** Inferred regulatory circuit of the 9 TFs. **(D)** ROC curve using training data from protein feature based method.

Due to technical issues, it is difficult to identify periodically expressed genes with low expression levels based on microarray [4,7]. We examined the expression levels of our 211 predicted genes. As shown in Figure 5A, compared to the positive set, these genes are lower in their mRNA expression levels [43] (p-value = 0.02, wilcoxon test; see Methods for details). To some extent, our genomic feature based method can complement traditional microarray methods and uncover lowly expressed cell cycle genes.

### Genomic feature based model reveals circular regulation of cell cycle transcription factors

The progression of cell cycle is under precise control of a serial of interrelated TFs. It has been proposed that cell cycle TFs regulate one another in a serial manner by forming a circular regulatory chain: a TF function in one phase will regulate TFs required for next phase [10]. Our genomic feature model recapitulates the conception. We selected the 9 key cell cycle TFs: Swi5, Ace2, Mbp1, Fkh1, Swi4, Swi6, Ndd1, Fkh2 and Mcm1, and investigated their genomic features, i.e. TF binding and motif existence, in their promoter regions. We examined the inter-regulatory relationship of the 9 key cell cycle TFs (Figure 5B). As shown in Figure 5C, there is a clear regulatory loop formed by these TFs. Mbp1 regulates the transcription of Swi4, which in turn regulates Ndd1 as inferred by motif existence. Ndd1, together with Fkh2 and/or Fkh1, promotes the transcription of Swi5.

TFs that regulates Mbp1 and Mcm1 is not clearly inferred from the TF binding data and motif information (Figure 5B). Mbp1 is periodically expressed, which might be regulated by other TFs or at the post-transcriptional level (e.g. mRNA degradation). Mcm1 is a key regulator at M/G1 checkpoint, but it is constantly expressed during the cell cycle. To achieve its function, Mcm1 cooperates with factors Yox1 and Yhp1p [18], which are activated by Mbp1 in G1 phase, and by Ndd1 and Fkh2 in G2 phase [10,18]. Yox1 and Yhp1p bind to positions upstream of Mcm1 binding sites acting as gene expression repressors (see Additional file 6 for more comprehensive circuit).

### Comparison with protein-feature based method

de Lichtenberg et al. have applied a machine-learning method that utilized protein features (such as phosphorylation and number of positively charged residues) to predict cell cycle genes [7]. We then asked which types of features (protein or genomic features) were more informative for cell cycle gene prediction. We applied our model to the same training data used by de Lichtenberg et al. The training data consists 97 high-confidence cell cycle genes and 556 non-cell cycle ones. To be consistent with their analysis, we also used 3 fold cross-validations to evaluate the performance of our method. As shown in Figure 5D, with this training data our genomic feature based model results in an AUC score of 0.921, which is significantly higher than that of the protein feature based method (AUC = 0.788). Detailed examination of model performance (Additional file 7) indicates that, as setting higher threshold, false positive rates dramatically decrease. Generally, genomic features are more effective for predicting periodical expression across the cell cycle than protein features.

In addition, we compared the two PLR models trained by the two different training data sets from de Lichtenberg et al. [7] and Cyclebase, respectively. First, the two models result in similar set of significant features. Specifically, the model using de Lichtenberg’s training data gives rise to a total of 7 significant features, among which 5 (Mbp1, M24, M25, M357, M46) are shared with the model using Cyclebase training data (Figure 2C). Secondly, we compared the top 211 most significant cell cycle genes predicted by the two models and found that 132 of them were overlapped. The genes in one of the top 211 lists are mostly predicted to have higher probabilities to be cell cycle ones in the other model.

## Discussion

The idea of modeling gene expression based on regulatory information has been applied to predict gene expression levels [14,44]. In this work, we used similar idea to quantify gene expression patterns assuming common mechanisms underlying cell cycle regulated genes. Genomic features used in our model include trans-regulators and cis-elements. They possess complementary effect in predicting cell cycle regulated genes. Trans-regulators’ binding gives the direct measure of involvement of transcription factors, whereas cis-elements give overall binding potentials of regulatory proteins, such as transcription factors and other proteins. Our method suggests cell cycle progression is possibly dominated by only a few key transcription factors and these transcription factors themselves form a regulatory circuit. Other cell cycle transcription factors may function downstream of these key regulators. Compared to previous protein feature based method, which focused on post-transcriptional level regulation, genomic feature based method demonstrates increased prediction performance. The improvement is not surprising as transcriptional level regulation is the first and the most critical step that controls gene expression. Even though our method could separate periodical genes from non-periodical ones with fair accuracy, cell cycle progression control is a much more complex system than could be simply explained by proximal transcription regulation alone. Other levels of regulation, such as distal transcription regulation, protein modification, activation, localization, and degradation are also important components. These regulations may account for false positives and false negative predictions in our method. Future works could focus on combining all kind of regulations together to explain cell cycle control.

One of the advantages of our method is the independence of microarray experiments in identifying novel periodical genes. As we known, microarray analysis is quite sensitive to de-synchronization and experimental condition influence. Also cell cycle process induced by different chemicals might harbor biases towards certain categories of cell cycle regulated genes. Thus, our method could provide an alternative way to complement and verify known list of cell cycle regulated genes. After quantifying cell cycle genes by general model, we also applied phase specific model to further classify genes. Phase specific prediction model is affected by the stage separation quality in Cyclebase.org. For example, several genes are classified having some ambiguity, as their expression peak lying in the conjunction of two stages. However, it could provide clues to stage specificity of genes and is easy to adapt to other predefined data.

Wu and Li proposed a simple method to predict cell cycle genes based on ChIP-chip, TF binding sites and other data [45]. They predicted 178 novel yeast cell cycles genes that were regulated by at least two of 17 cell cycle TFs. Here we applied a supervised statistical model to improve their method. Out of the 178 cell cycle genes predicted by Wu et al., 8 are in our positive training set. In the remaining 170 genes, 49 are also predicted to be cell cycle genes by our model (i.e. in the 211 gene list). Out of the 170 genes, 69% (118) have physical interactions with known cell cycle genes, and 31% (52) have at least one of the 9 supporting evidences (see Methods), compared with 63% (132) and 40% (85) for the 211 cell cycle genes predicted by our model. GO analysis indicates that the 170 genes were enriched in various metabolic processes (e.g. glucose metabolic process), while the 211 genes we predicted are more involved in cell cycle related processes (e.g. cell wall and chromosome condensation).

There are also certain drawbacks of our model. (1) Different transcription factors or motifs are weighted the same in our model, without considering their position and orientation relative to the gene. This information may further influence the expression peak time of genes. (2) As we shown, different cell cycle stages possess intrinsic varied regulatory mechanisms, but the general model we used assumes all cell cycle regulated genes harbor common genomic features. Thus the model could be further modified to accommodate both common features and stage specific features. Our genomic-based method can also be modified and extended to predict genes in other periodical processes, such as human cell cycle process and circadian phenomenon, or generally to transcriptionally regulated processes, such as tissue-specific expression.

## Conclusions

We model the cell cycle regulated genes using their genomic features (transcription factor binding and motif profiles), especially in the promoter regions. It suggests that cell division, to a large extent, is controlled by a relatively small number of master transcription factors. These master TFs form a self-regulatory circuit and then regulate downstream TFs and genes. Genomic feature based method exempts us from the drawbacks of microarray and helps us to reveal the regulatory machinery. This concept can be applied to other organisms and transcriptionally regulated processes.

## Methods

### Cell cycle and non-cell cycle regulated genes

In Cyclebase, Gauthier et al. collected 6 cell cycle time course microarray datasets and performed a meta-analysis to calculate the significance of gene periodicity. The resulting p-values are based on a summarization of all these 6 datasets, which represent a more confident evaluation of periodical expression than a single dataset. Based on Cyclebase, we select 599 cell cycle genes that show significant periodical expression pattern (combined p-value < 0.001). At the same time, we select 454 non-cell cycle genes that are not periodically expressed in any of the 6 datasets (p-value > 0.1). Alternative, we also tried another non-cell cycle gene set by randomly selecting 454 genes that are not in the positive set. Classification models based on the two sets of training data achieve similar classification accuracy and consistent results.

### ChIP-chip data for yeast TFs

We use the large-scale ChIP-chip experiments performed by Harbison et al. to determine the TF-gene binding strength. The data contains binding information of 203 yeast TFs in the promoter region (the DNA sequences from translation initiation site up to 1 kb upstream) of all yeast genes under YPD condition. Each TF-gene pair is assigned an occupancy ratio, which reflects binding strength of the TF to the promoter of the gene. A larger occupancy ratio indicates stronger binding strength. We represent the ChIP-chip occupancy ratio data as a matrix with 6,229 rows and 203 columns, each row corresponding to a gene and each column corresponding to a TF. These TF binding features are used as predictors in our model to classify cell cycle gene versus non-cell cycle ones.

### Motif matching scores for all yeast genes

In a systematic analysis performed by Beer et al., 666 potential regulatory motifs are enriched in the promoter regions (the DNA sequences from translation initiation site up to 800 bp upstream) of all yeast genes using the AlignACE software. The occurrences of each motif in the promoter region of all genes were then determined by setting up a threshold of matching-score larger than 0.5. From this motif discovery data, we select 537 motifs after removing redundancy. For 46 of these motifs, the associated transcription factors have been determined according to literatures.

Based on this data, we define a matching-score matrix, which contains 6,328 rows each corresponding to a yeast gene, and 539 columns each corresponding to a putative motif. The element in the matrix is the aggregated matching-score of a motif in the promoter region of a gene. Namely, when a motif occurs with multiple copies in a promoter, we calculate the summation of all the matching-scores. If no occurrence is found in the promoter of a gene, the score is set to 0. The aggregated matching-score reflects the binding affinity of a motif to the promoter of a gene. These motif features are also used as predictors in our model.

For a set of selected motifs without corresponding transcription factors, we used TOMTOM [26] to quantify motif similarity with known transcription factors. Transcription factor identified with highest similarity score and with p-value smaller than 0.01 is assigned as the potential TF for that motif.

### Penalized logistic regression models and other classification methods

We apply the penalized logistic regression (PLR) method to classify yeast genes into cell cycle and non-cell cycle ones. To train the model, a positive and a negative gene set are prepared based on the Cyclebase as described above. From the TF binding and motif features, we pre-select those that are significantly different between the cell cycle and the non-cell cycle gene set (p-value <0.001, the student t-test), which are used as predictors.

PLR imposes a penalty term to each correlation coefficient to overcome the colinearity among variables and the over-fitting problem. Specifically, we use quadratic penalization term (L2 norm) in this analysis. For a gene *i*, we denote its predictor vector as *x*_*i*_, and *y*_*i*_ = 1 if it is a cell cycle gene and *y*_*i*_ = 0 otherwise. The logistic regression model can be defined as: $\log\frac{\mathrm{Pr}\left(y=1|x\right)}{\mathrm{Pr}\left(y=0|x\right)}=\beta_{0}+x^{t}\beta$, where ***β*** is the parameter vector to be estimated (*β* = (*β*_1_, *β*_2_, …, *β*_*m*_)^*t*^ for the model with ***m*** predictors).

Given this model, we can write the probability $p_{i}=\mathrm{Pr}\left(y_{i}=1\right)=\frac{\exp\left(\beta_{0}+x^{t}\beta \right)}{1+\exp\left(\beta_{0}+x^{t}\beta \right)}$. PLR estimates the parameter vector *θ* = (*β*_0_, *β*)^*t*^ by minimizing the following equation: $L\left(\beta_{0},\beta ,\lambda \right)=-l\left(\beta_{0},\beta \right)+\frac{\lambda }{2}||\beta |{|}_{2}^{2}$, where the first term *l* indicates the binomial log-likelihood, $||\beta |{|}_{2}^{2}$ is the quadratic penalization term, and λ is a positive constant. Prediction performance with varied λ is shown in Additional file 8.

In addition to the PLR method, we also test other supervised machine-learning approaches for predicting cell cycle genes, including SVM (support vector machine), Random forest and regular logistic regression. The classification accuracy of these methods is evaluated by calculating the AUC score, area under ROC (receiver operating characteristic) curve, based on 10 fold cross-validation results. The best accuracy is achieved by PLR method. To implement these methods, the R packages, “stepPlr”, “e1071”, “randomForest”, “glm” are used for PLR, SVM, Random Forest, and regular logistic regression respectively.Multinomial logistic regression analysis (RMLR) extends PLR to multiple categories classification, assigning each category relative possibility among all possible results. RMLR follows the similar schema as PLR. Instead of using t-test, one-way ANOVA is conducted to pre-select features (p < 0.01). MATLAB package “logregFit” is used to implement the RMLR method. For multiple class classification, AUC score is the area under ROC surface. We follow Hand and Till’s multi-class AUC score calculation [32]. $\mathrm{AU}C_{\mathrm{total}}=\frac{2}{|C|\left(|C|-1\right)}\sum_{\left\{C_{i},C_{j}\right\}\in C}\mathrm{AUC}\left(C_{i},C_{j}\right)$, with *C* are the different categories.

### Enrichment of protein-protein interactions

We download yeast protein-protein interaction data from BIOGRID [33]. Among the 211 predicted genes, 132 have physical interaction with at least one of the 599 known cell cycle genes in the positive training set. There are 126,389 possible pairs between the predicted cell cycle genes and the known cell cycle genes, among which 499 pairs have physical interactions. Meanwhile, we observed 9,943 physical interactions out of the 2,969,243 possible pairs between the predicted non-cell cycle genes and the known cell cycle genes. We used the Fisher’s exact test to calculate the significance for physical interaction enrichment of predicted cell cycle genes with known cell cycle genes. Specifically, the R function “fisher.test” is implemented for the computation.

### Gene ontology analysis

We perform gene ontology (GO) analysis by using the bioinformatics tool DAVID (the Database for Annotation, Visualization and Integrated Discovery) [39]. We note that we exclude all genes from the positive and negative gene sets, and use the remaining genes as the background gene set for GO analysis to avoid the impact of known cell cycle genes. Function annotation of yeast genes is obtained from the Saccharomyces Genome Database (SGD) [38]. To cluster gene function, we use the GO slim mapper in SGD.

### Comparison of our predictions with results from previous cell cycle studies

To find supporting evidence for our prediction, we collect 9 different datasets: protein-feature based method prediction [7], known cell cycle regulated genes [7], datasets from Zhao [6], Spellman [3], Cho [2], Granovskaia [34] and Rowicka [35], weakly expressed periodical gene paper [4] and 6 experiments in Cyclebase.org. In total, 85 out of 211 genes have evidence from these resources.

### Normalization of TF binding and motif matching scores

The regulatory potential of gene by a TF can be reflect by the TF binding score from ChIP-chip experiment or by the motif matching score from sequence analysis. To make the TF binding scores and motif scores directly comparable, we combine the TF binding matrix and motif-matching matrix, and perform quantile normalization to normalize them. For each feature (740 TF or motif features), the values are sorted across all genes and then set to the average of distributions. So the highest value in all genes becomes the mean of the highest values, the second highest value becomes the mean of the second highest values, and so on. After quantile normalization, the TF binding and motif score features will have the same distribution and are comparable with one another.

We use the following criteria to determine the TF-TF regulatory relationships based on TF binding or motif scores. (1) quantile normalization score for TF binding or motif is greater than 0.9; (2) for TF binding data, the log transformed occupancy ratio is larger than 2; (3) for motif data, the motif score is larger than 0.5.

### Availability of supporting data

All the supporting data are included as additional files. The related files are also available at: http://archive.gersteinlab.org/proj/yeastCC/.

## Competing interests

The authors declare that they have no competing interests.

## Authors’ contributions

CC, YF and MG designed the study. CC, YF and LS carried out the studies. CC, YF and MG drafted the manuscript. All the authors read and approved the final manuscript.

## Supplementary Material

Additional file 1 Comparison of predicted gene lists from different methods (cut-off = 0.8). Click here for file

Additional file 2 **211 predicted cell cycle regulated genes.** Each gene is annotated with general and phase specific prediction scores and corresponding gene functions. Click here for file

Additional file 3 211 genes ordered by support from other studies. Click here for file

Additional file 4 GO analysis of newly predicted genes. Click here for file

Additional file 5 Expression profiles of Cla4 in Cyclebase. Click here for file

Additional file 6 **Extended circuit of transcription factors.** a) Normalization scores of TFs respect to key genomic features in cell cycle regulation. b) Regulatory circuit of transcription factors. “Red” lines are regulatory relationship between key transcription factors. “Green” lines are regulatory relationships between key TFs and other TFs. The inhibitory effects are from literatures (the effect is on function level instead of promoter regulation level). Yox1 and Yhp1 are under positive regulation of several TFs, such as Mbp1, Fkh2 and Ndd1. Their expression will inhibit Mcm1 function in G1, S and G2 phases. It is interesting to notice that in M phase Mcm1 promotes Yox1 and Yhp1 expression, and at the same time, Yox1 and Yhp1 inhibit Mcm1’s function. Click here for file

Additional file 7 Prediction performance with training data from de Lichtenberg et al. Click here for file

Additional file 8 **AUC score changes with respect to penalized term λ in TF + Motif model (cell cycle genes vs. non cell cycle genes).** AUC score is quite insensitive to λ in certain ranges. Thus, our model is quite stable to parameter λ. Click here for file
