# Supplementary figures and images for: Identification of yeast cell cycle regulated genes based on genomic features
Source: BMC Syst Biol. 2013 Jul 29;7:70. doi: 10.1186/1752-0509-7-70 (PMC3734186; doi:10.1186/1752-0509-7-70)

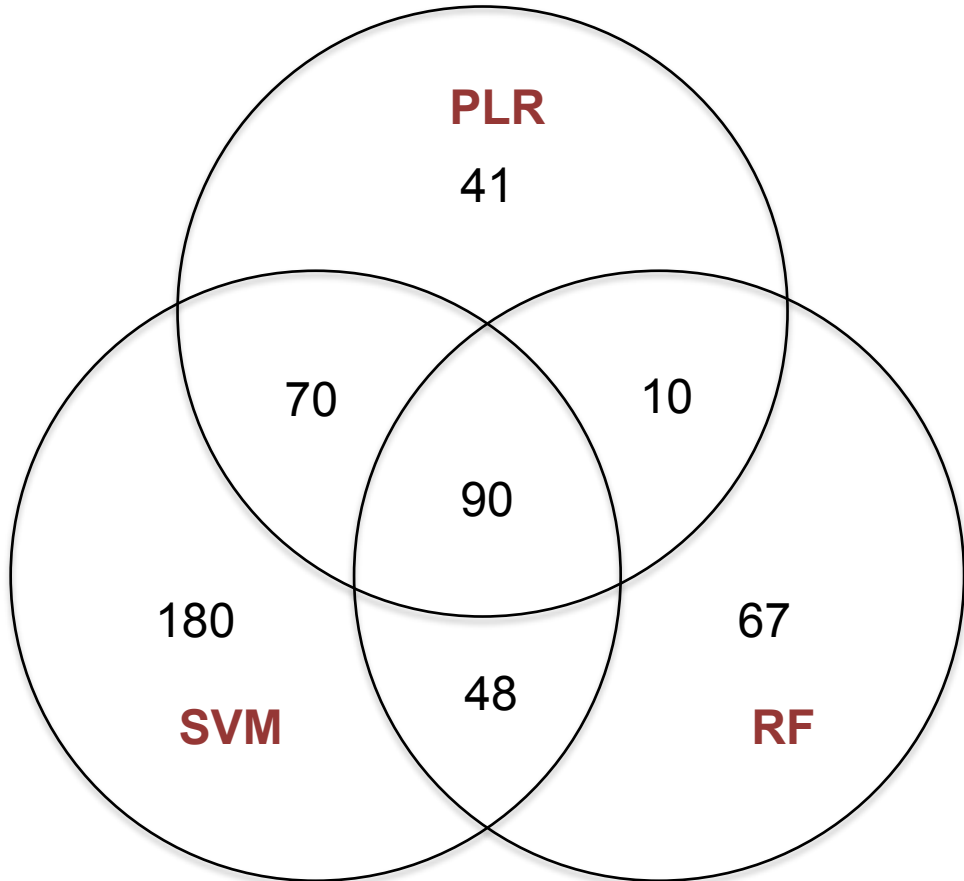

Supplement: Additional file 1 — Comparison of predicted gene lists from different methods (cut-off = 0.8). [file 1752-0509-7-70-S1.pdf]

# CLA4

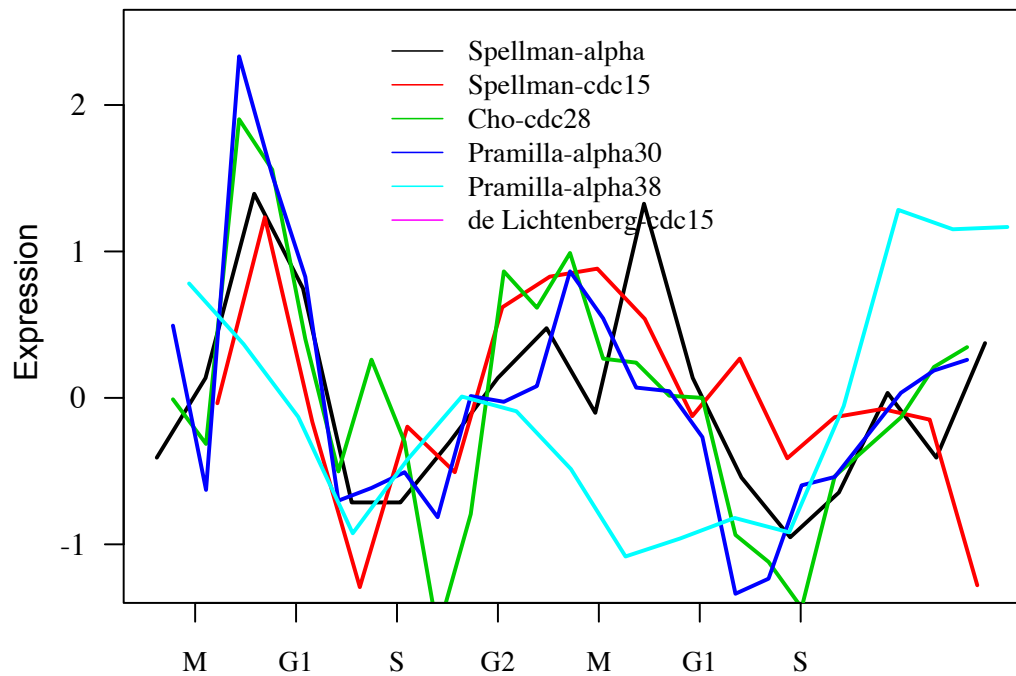

Supplement: Additional file 5 — Expression profiles of Cla4 in Cyclebase. [file 1752-0509-7-70-S5.pdf]

**A**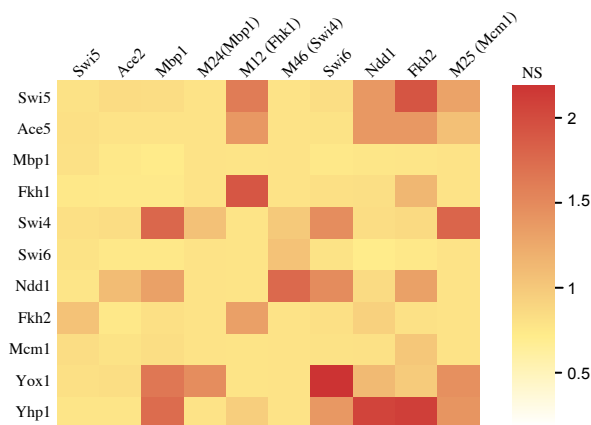**B**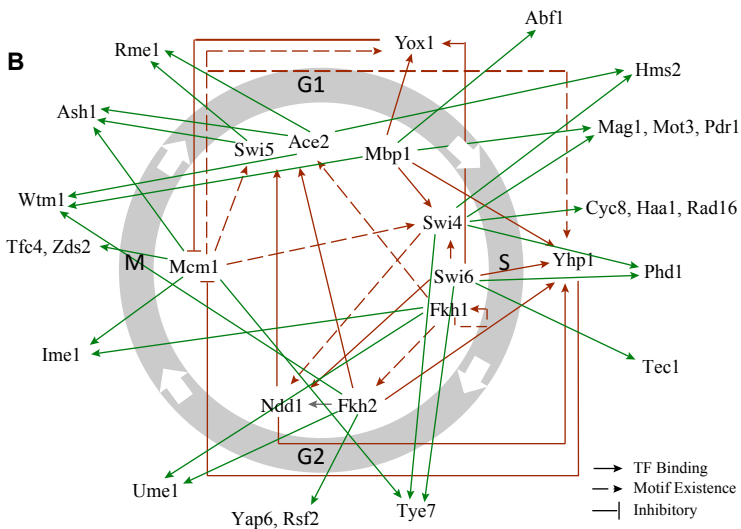

Supplement: Additional file 6 — Extended circuit of transcription factors. a) Normalization scores of TFs respect to key genomic features in cell cycle regulation. b) Regulatory circuit of transcription factors. “Red” lines are regulatory relationship between key transcription factors. “Green” lines are regulatory relationships between key TFs and other TFs. The inhibitory effects are from literatures (the effect is on function level instead of promoter regulation level). Yox1 and Yhp1 are under positive regulation of several TFs, such as Mbp1, Fkh2 and Ndd1. Their expression will inhibit Mcm1 function in G1, S and G2 phases. It is interesting to notice that in M phase Mcm1 promotes Yox1 and Yhp1 expression, and at the same time, Yox1 and Yhp1 inhibit Mcm1’s function. [file 1752-0509-7-70-S6.pdf]

Prediction Performance

— Sensitivity  
— False Positive Rate

0.5

0

0

0.5

1

Threshold

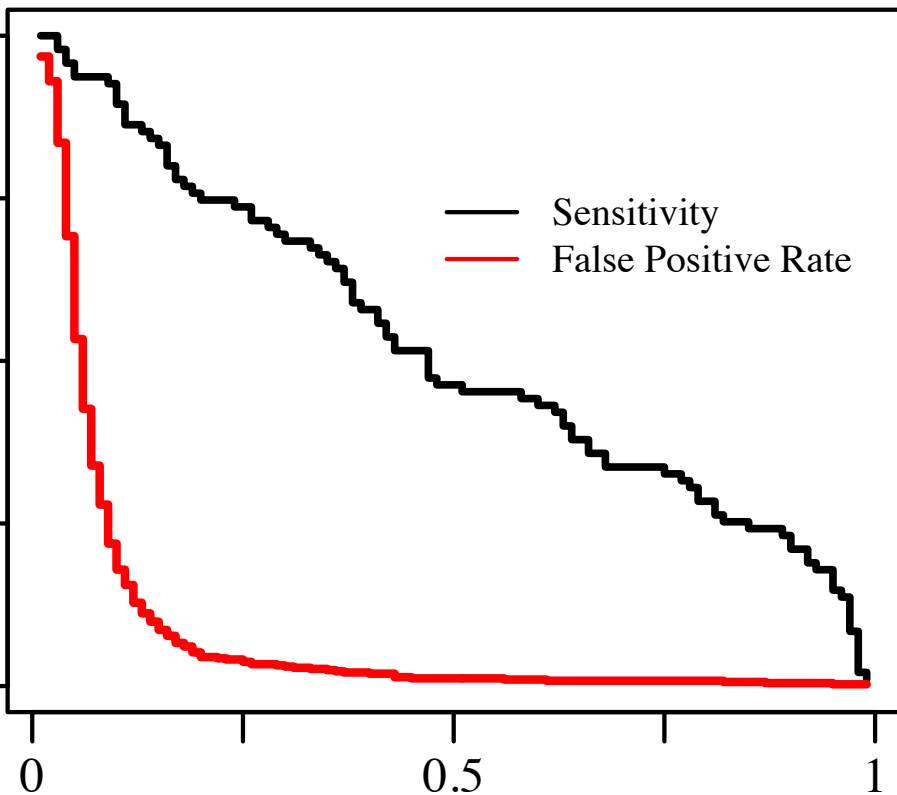

Supplement: Additional file 7 — Prediction performance with training data from de Lichtenberg et al. [file 1752-0509-7-70-S7.pdf]

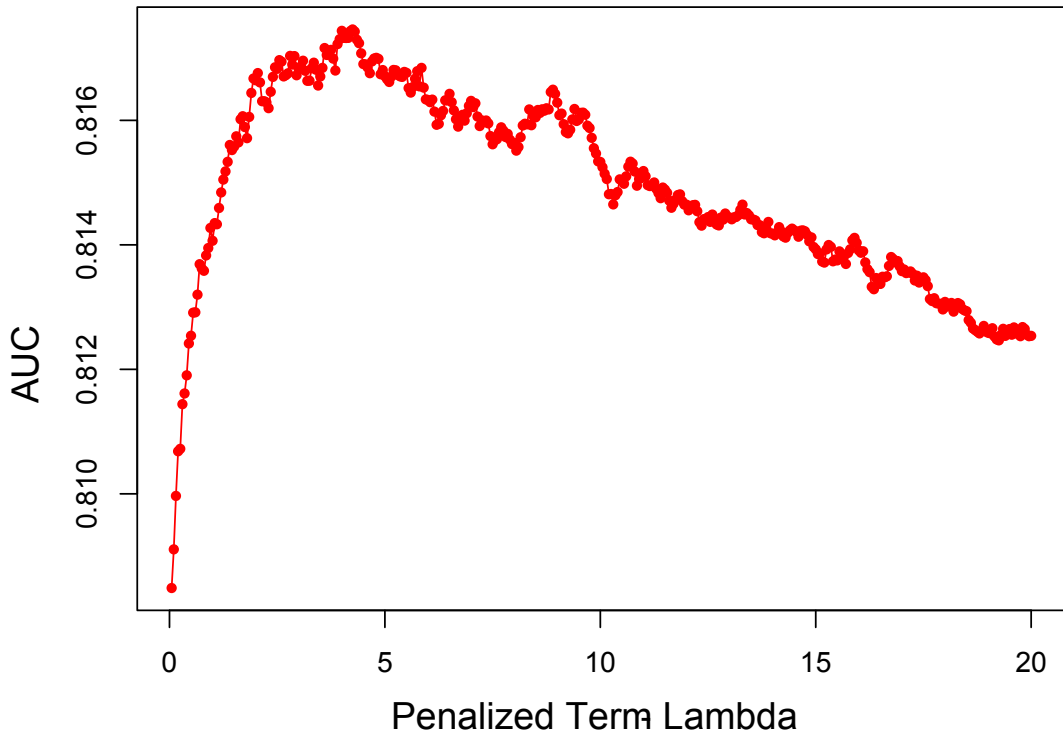

Supplement: Additional file 8 — AUC score changes with respect to penalized term λ in TF + Motif model (cell cycle genes vs. non cell cycle genes). AUC score is quite insensitive to λ in certain ranges. Thus, our model is quite stable to parameter λ. [file 1752-0509-7-70-S8.pdf]
